# A space network structure constructed by tetraneedlelike ZnO whiskers supporting boron nitride nanosheets to enhance comprehensive properties of poly(L-lacti acid) scaffolds

**DOI:** 10.1038/srep33385

**Published:** 2016-09-15

**Authors:** Pei Feng, Shuping Peng, Ping Wu, Chengde Gao, Wei Huang, Youwen Deng, Cijun Shuai

**Affiliations:** 1 Hunan Provincial Tumor Hospital and the Affiliated Tumor Hospital of Xiangya School of Medicine, School of Basic Medical Science, Central South University, Changsha 410013, China; 2 State Key Laboratory of High Performance Complex Manufacturing, the State Key Laboratory for Powder Metallurgy, Central South University, Changsha 410083, China; 3 State Key Laboratory of Solidification Processing, Northwestern Polytechnical University, Xi’an 710072, China; 4 School of Basic Medical Science, Central South University, Changsha 410078, China; 5 College of Chemistry, Xiangtan University, Xiangtan 411105, China; 6 Department of Orthopedics, The Second Xiangya Hospital, Central South University, Changsha 410011, China

## Abstract

In this study, the mechanical strength and modulus of poly(L-lacti acid) (PLLA) scaffolds were enhanced with the mechanical properties of boron nitride nanosheets (BNNSs) and tetraneedlelike ZnO whiskers (T-ZnO_w_). The adhesion and proliferation of cells were improved as well as osteogenic differentiation of stem cells was increased. Their dispersion statues in PLLA matrix were improved through a space network structure constructed by three-dimensional T-ZnO_w_ supporting two-dimensional BNNSs. The results showed that the compressive strength, modulus and Vickers hardness of the scaffolds with incorporation of 1 wt% BNNSs and 7 wt% T-ZnO_w_ together were about 96.15%, 32.86% and 357.19% higher than that of the PLLA scaffolds, respectively. This might be due to the effect of the pull out and bridging of BNNSs and T-ZnO_w_ as well as the crack deflection, facilitating the formation of effective stress transfer between the reinforcement phases and the matrix. Furthermore, incorporation of BNNSs and T-ZnO_w_ together into PLLA scaffolds was beneficial for attachment and viability of MG-63 cells. More importantly, the scaffolds significantly increased proliferation and promoted osteogenic differentiation of human bone marrow mesenchymal stem cells (hBMSCs). The enhanced mechanical and biological properties provide the potentials of PLLA/BNNSs/T-ZnO_w_ scaffolds for the application into bone tissue engineering.

Poly(l-lacti acid) (PLLA) is an attractive scaffold material owing to its biocompatibility and processability, and tunable biodegradability^1^,^2^,^3^. It can completely degrade to carbon dioxide and water by hydrolysis. In addition, PLLA has biological interactions with host cells and can support bone regeneration after implantation. However, the poor mechanical properties hamper its application in the repair of load bearing bone defects^4^,^5^. Introduction of nano second phase such as nanoparticles, nanotubes, nanosheets and whiskers into polymer matrix is a promising approach to overcome the defects^6^,^7^,^8^,^9^,^10^. While the enhancing efficiency by these nano second phases in polymers is limited owing to agglomeration^11^,^12^,^13^. In very recent years, the hybridization of one-dimensional nanotubes and two-dimensional nanosheets (or nanoplatelets) has been proposed to promote the dispersion^14^,^15^,^16^,^17^,^18^. While carbon nanotubes are easily attached in the direction paralleling to graphene surface because of the strong π-π stacking interaction between the two materials.

Herein, a space network structure is constructed by three-dimensional tetraneedlelike ZnO whiskers (T-ZnO_w_) in cooperation with boron nitride nanosheets (BNNSs). In this space network structure, T-ZnO_w_ can support BNNSs in the direction perpendicular to the surface, which is beneficial to the dispersion of each other more effectively. Boron nitride (BN), so-called “white graphite”, is a two-dimensional layered material that exhibits a hexagonal crystal structure. BNNSs possess elastic modulus (505–1031 GPa) and tensile strength (>150 GPa), which provides them potential reinforcement for strengthening of polymer scaffold^19^,^20^. Another important consideration for using BNNSs in bone tissue engineering is their good biocompatibility. Previous studies have shown that BN exhibited non-cytotoxic and good cytocompatibility to osteoblasts, macrophages, human embryonic kidney cells and neuroblastoma cells^21^,^22^,^23^,^24^. In addition, it could promote the differentiation of mesenchymal stem cells (MSCs) into osteoblasts^25^.

T-ZnO_w_, a new kind of whiskers with a single crystal, have been used as an ideal reinforcement to improve the mechanical properties of polymer because of the super high strength and modulus^26^,^27^. They exhibit needle-like tetrapod shapes with four needle-like arms extending from the same center in four directions in three-dimensional space, which leads to a homogeneous stress distribution in the polymer matrix^28^. Niu *et al.* added T-ZnO_w_ to resin composite and found that the addition of T-ZnO_w_ could improve the flexural, compressive and tensile strength of resin composite^29^. Apart from this, ZnO was biocompatible and could promote cell adhesion and growth^30^,^31^. Additionally, some studies reported that ZnO had bioactive and could facilitate apatite formation after soaking in simulated body fluid (SBF)^32^,^33^.

Therefore, besides the constructing of the space network structure to promote dispersion in PLLA matrix, there are another two aims for using of BNNSs and T-ZnO_w_ together, namely: (i) to enhance the compressive strength, modulus and Vickers hardness of PLLA scaffold with the advantage of their mechanical properties, and (ii) to improve the cell attachment, proliferation and differentiation abilities with the advantage of their biological properties.

## Results

**Figure 1:**
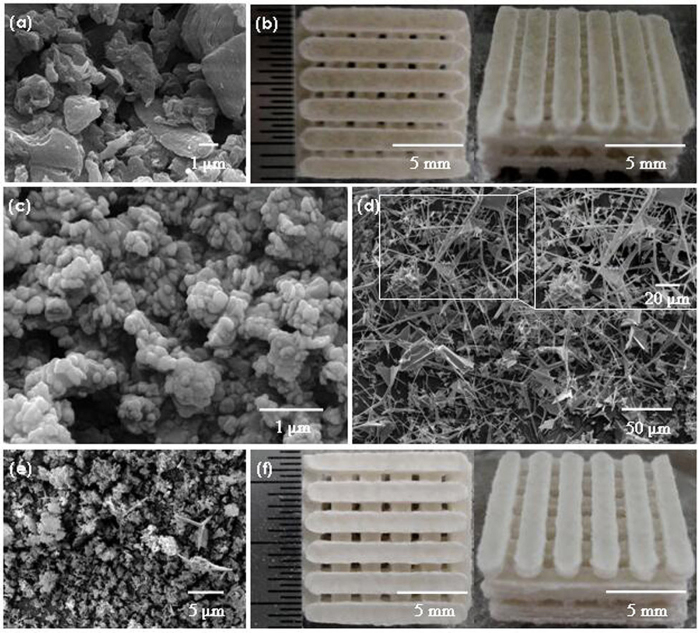
SEM and Optical images. SEM images for (**a**) PLLA powder, (**c**) BNNSs powder, (**d**) T-ZnO_w_ powder, (**e**) Composite powder, and Optical images for the (**b**) PLLA scaffold and (**f**) PLLA/BN/ZnO scaffold.

The images for the original powder, PLLA scaffold and PLLA/BN/ZnO scaffold are shown in Fig. 1. PLLA powder have irregular shape with the particle size from 0.2 to 5 μm (Fig. 1a). BNNSs powder has platelet form and the morphology is uniform (Fig. 1c). T-ZnO_w_ powder has tetraneedlelike nanostructure, and the four needlelike arms extend from the same center in four directions (Fig. 1d). The BNNSs and T-ZnO_w_ powder were randomly dispersed with PLLA powder, and individual T-ZnO_w_ was clearly seen and maintained its original morphology in composite powder after mixing (Fig. 1e). The composite powder was sintered on a SLS system for fabricating porous scaffolds, and the sintering process was described in our previous study^34^. All the scaffolds had uniform macropores with interconnected pore channels and the pore size was around 800 μm (Fig. 1b,f). The size of scaffolds was about 13.5 mm × 13.5 mm × 7 mm and there was no significant difference between the PLLA scaffold and the PLLA/BN/ZnO scaffold.

**Figure 2 f2:**
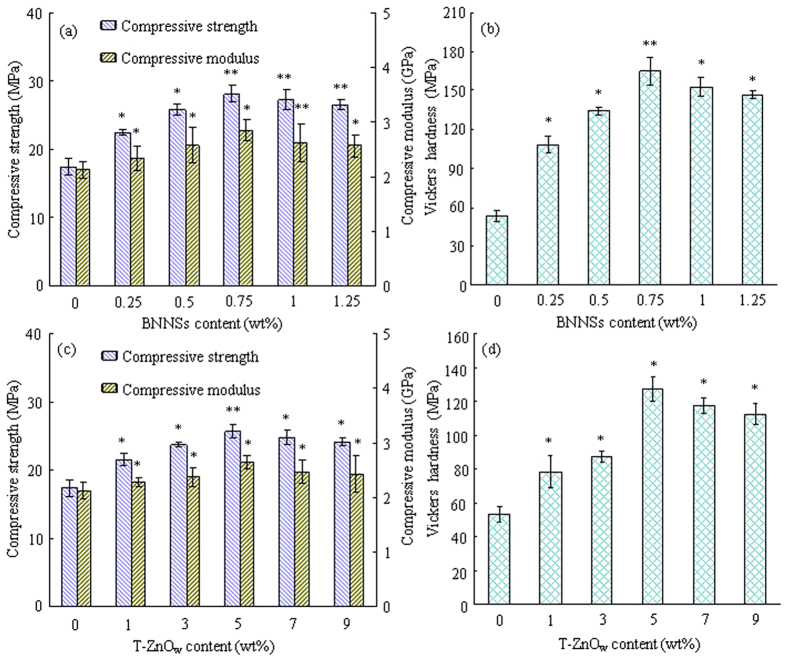
The analysis of compressive strength, modulus and Vickers hardness of scaffolds. (**a,b**) PLLA/BN scaffolds; (**c,d**) PLLA/ZnO scaffolds. *Represents significant difference (*p* < *0.05*) and **represents very significant difference (*p* < *0.01*) when the PLLA/BN or PLLA/ZnO scaffolds, as compared to the PLLA scaffolds.

Mechanical properties (compressive strength, modulus and Vickers hardness) of the scaffolds with BNNSs or T-ZnO_w_ at different content were measured (Fig. 2). The compressive strength, modulus and Vickers hardness first increased with BNNSs content increasing from 0 to 0.75 wt% and then decreased with BNNSs content further increasing to 1.25 wt% (Fig. 2a,b). The highest compressive strength, modulus and Vickers hardness were 28.17 MPa, 2.85 GPa and 164.76 MPa, respectively. The compressive strength, modulus and Vickers hardness of the PLLA/ZnO scaffolds increased from 17.41 MPa, 2.13 GPa and 53.19 MPa to 25.72 MPa, 2.64 GPa and 127.71 MPa with T-ZnO_w_ content increasing from 0 to 5 wt%, respectively (Fig. 2c,d). And then decreased to 24.16 MPa, 2.43 GPa and 112.75 MPa with T-ZnO_w_ content further increasing to 9 wt%.

**Figure 3 f3:**
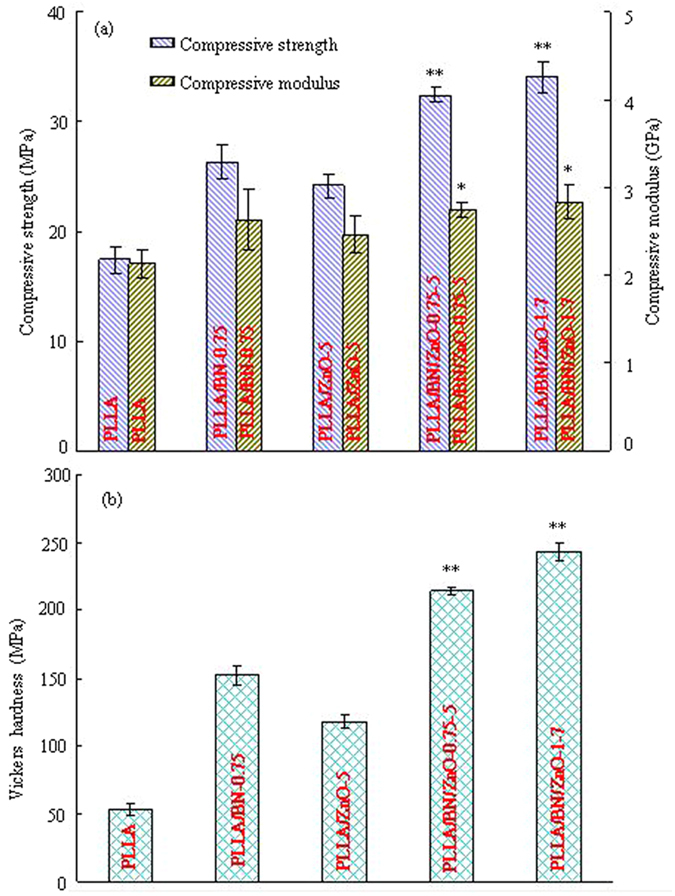
The compressive strength and modulus, and Vickers hardness of the scaffolds. (**a**) Compressive strength and modulus; (**b**) Vickers hardness. *Represents significant difference (*p* < *0.05*) and **represents very significant difference (*p* < *0.01*) when the PLLA/BN/ZnO scaffolds, as compared to the PLLA, PLLA/BN-0.75 and PLLA/ZnO-5 scaffolds.

The mechanical properties of the PLLA, PLLA/BN-0.75, PLLA/ZnO-5, PLLA/BN/ZnO-0.75-5 and PLLA/BN/ZnO-1-7 scaffolds are shown in Fig. 3. The PLLA/BN/ZnO-0.75-5 scaffolds had higher compressive strength, modulus and Vickers hardness than the PLLA, PLLA/BN-0.75 and PLLA/ZnO-5 scaffolds, which indicated that the addition of BNNSs and T-ZnO_w_ together in the PLLA matrix resulted in the higher mechanical properties than that of addition of BNNSs or T-ZnO_w_ individually (*p* < *0.01*). The compressive strength, modulus and Vickers hardness of the PLLA/BN/ZnO-1-7 scaffolds were higher than that of the PLLA/BN/ZnO-0.75-5 scaffolds and about 96.15%, 32.86% and 357.19% higher than that of the PLLA scaffolds, respectively.

**Figure 4 f4:**
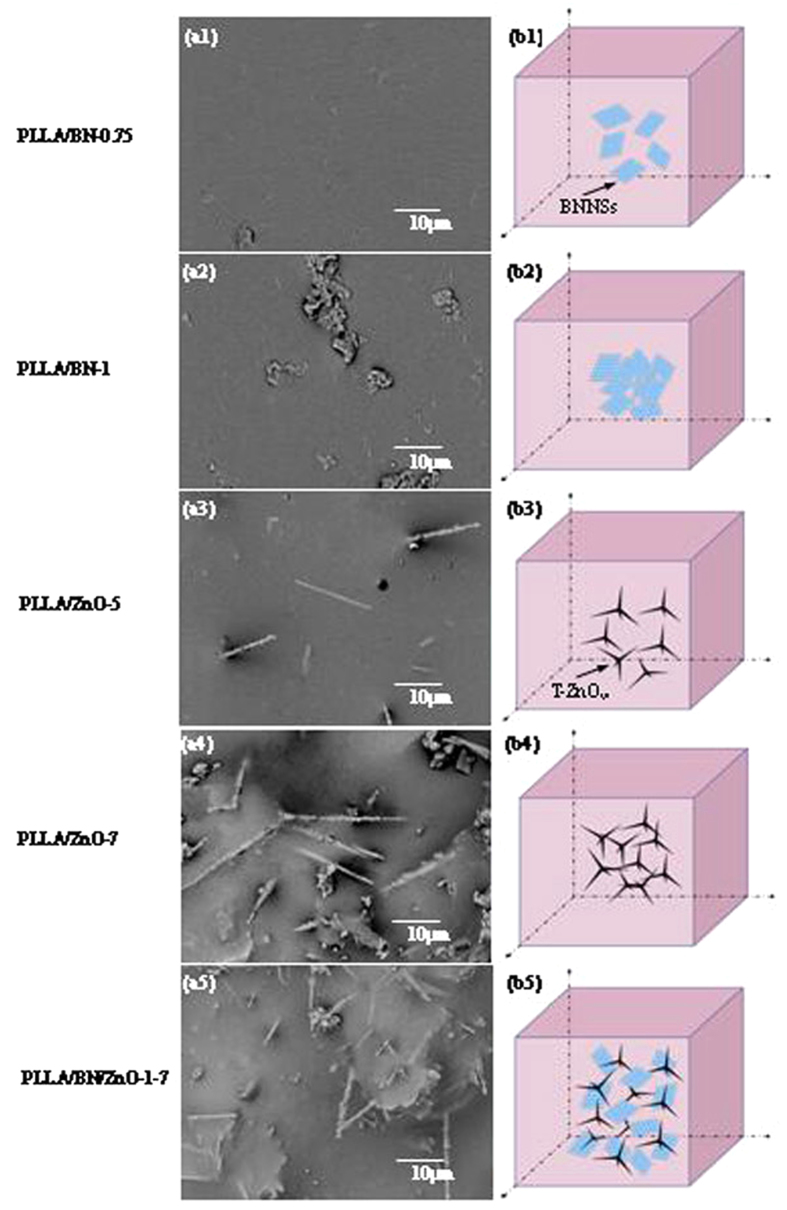
The morphologies and corresponding schematic. The morphologies (**a1–a5**) of the scaffolds under SEM and the corresponding schematic (**b1–b5**) of BNNSs and T-ZnO_w_ dispersion in the PLLA matrix.

The surface morphologies and the corresponding schematic of BNNSs and T-ZnO_w_ dispersion in the PLLA matrix are shown in Fig. 4. A homogeneous dispersion of BNNSs throughout the PLLA matrix was achieved at lower BNNSs content, such as 0.75 wt% (Fig. 4a1,b1). While part of the BNNSs aggregated at higher content (1 wt%) because of the great specific surface area (Fig. 4a2,b2). For the PLLA/ZnO scaffolds, T-ZnO_w_ were well dispersed in the matrix when the content was 5 wt% (Fig. 4a3,b3), while further increased the content led to the poor dispersion due to the strong van der Waals interactions (Fig. 4a4,b4). The poor dispersion of BNNSs and T-ZnO_w_ in PLLA matrix would result in some defects on the scaffolds surface, which decreased the overall mechanical properties^35^,^36^,^37^. However, the dispersion state of BNNSs and T-ZnO_w_ in the matrix was improved by incorporation of BNNSs and T-ZnO_w_ together into PLLA matrix (Fig. 4a5,b5). The reason was that two-dimensional BNNSs and three-dimensional T-ZnO_w_ could construct a space network structure which exhibited a synergetic enhancing effect on the mechanical properties of scaffolds. In the structure, T-ZnO_w_ could support the BNNSs in the direction perpendicular to the surface, which might be beneficial to the dispersion of each other more effectively. This structure could be further observed from the fracture images of the scaffolds.

**Figure 5:**
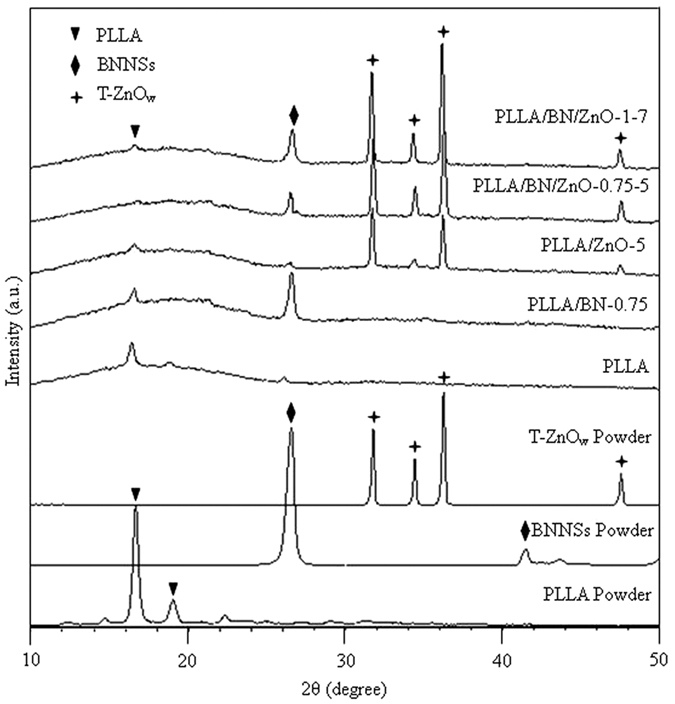
XRD spectra of PLLA, BNNSs and T-ZnO_w_ powder, and the PLLA, PLLA/BN-0.75, PLLA/ZnO-5, PLLA/BN/ZnO-0.75-5 and PLLA/BN/ZnO-1-7 scaffolds.

The XRD spectra of the PLLA, PLLA/BN, PLLA/ZnO and PLLA/BN/ZnO scaffolds are shown in Fig. 5. The results of the original PLLA, BNNSs and T-ZnO_w_ powder were also provided for comparison. It could be seen that PLLA powder had two diffraction peaks at 2θ = 16.3° and 18.6°, while the diffraction peaks of BNNSs and T-ZnO_w_ were 26.8°, 41.8°, and 31.8°, 34.4°, 36.3°, 47.5°, respectively. The peaks of other impurity phases were not found. In addition to the stacked peaks of PLLA and BNNSs, no other peaks were observed in the PLLA/BN-0.75 scaffolds. Similarly, no new peak was found in the PLLA/ZnO-5 scaffolds either. The pattern of PLLA/BN/ZnO-0.75-5 and PLLA/BN/ZnO-1-7 scaffolds still retained the same profile as observed in the PLLA scaffolds, and the additional diffraction peaks at 26.8°, 41.8°, and 31.8°, 34.4°, 36.3°, 47.5° corresponded to BNNSs and T-ZnO_w_, respectively. These results indicated that BNNSs and T-ZnO_w_ still existed and there was no new phase formed in the PLLA/BN, PLLA/ZnO and PLLA/BN/ZnO scaffolds after laser sintering.

**Figure 6:**
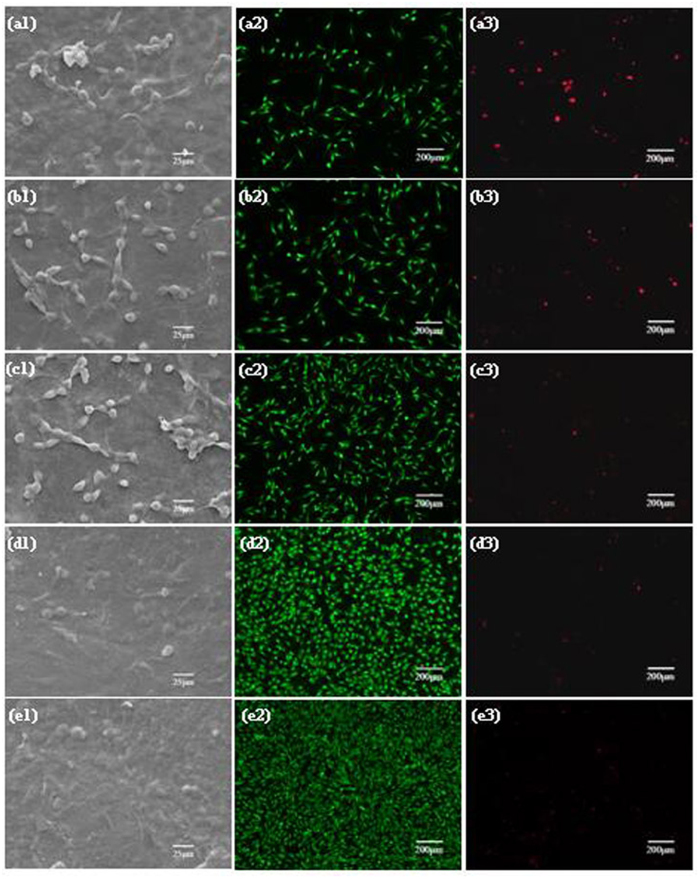
The viability of MG-63 cells on the scaffolds after 7 days of culture. (**a1–a3**) PLLA scaffolds; (**b1–b3**) PLLA/BN-0.75 scaffolds; (**c1–c3**) PLLA/ZnO-5 scaffolds; (**d1–d3**) PLLA/BN/ZnO-0.75-5 scaffolds; (**e1–e3**) PLLA/BN/ZnO-1-7 scaffolds; **(a1,b1,c1,d1,e1**) the SEM images; **(a2,b2,c2,d2,e2**) live cells were stained green; **(a3,b3,c3,d3,e3**) dead cells were stained read. The fluorescence images were acquired using the same camera at the same level of excitation light.

To evaluate the response of cells to the scaffolds, MG-63 cells were seeded and cultured onto the PLLA, PLLA/BN-0.75, PLLA/ZnO-5, PLLA/BN/ZnO-0.75-5 and PLLA/BN/ZnO-1-7 scaffolds for 7 days, and their adhesion, viability and proliferation were studied (Fig. 6). MG-63 cells could adhere to all types of scaffolds, indicating the good cytocompatibility. More cells attached on the PLLA/BN and PLLA/ZnO scaffolds than on the PLLA scaffolds (Fig. 6a1), and some cells started to spread out with cytoplasmic bridges between them (Fig. 6b1,c1). The cells were spreading well and almost completely covered the PLLA/BN/ZnO scaffolds surface, especially the PLLA/BN/ZnO-1-7 scaffolds (Fig. 6d1,e1). In the fluorescence images, live cells were stained green, while dead cells were stained red, respectively. The cells were uniformly distributed on all the scaffolds, indicating the scaffold materials were homogenous. They grew well on the PLLA/BN/ZnO scaffolds and spread to the whole areas of the scaffold surface, while the PLLA scaffolds had the lowest number of live cells. The increased cell attachment and viability of the PLLA/BN/ZnO scaffolds suggested that the addition of BNNSs and T-ZnO_w_ had positive effect on cell response.

**Figure 7:**
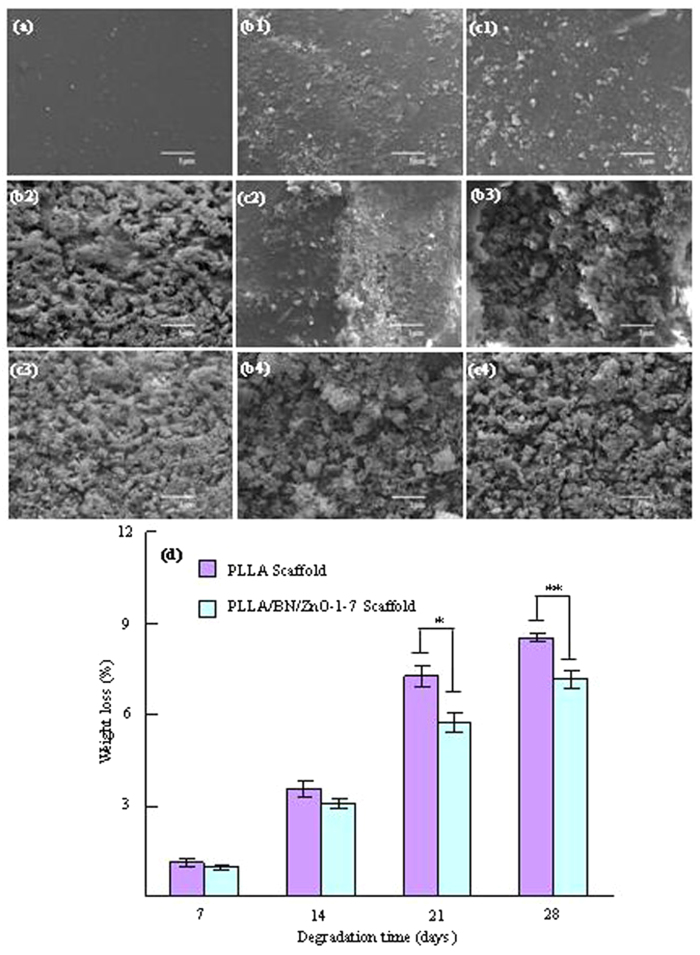
The morphologies of the scaffolds before and after soaked in PBS and weight loss. SEM images (**a,b1,c1, b2,c2,b3,c3,b4,c4**) and weight loss (**d**) of the PLLA (**a,b1,b2,b3,b4**) and PLLA/BN/ZnO-1-7 (**a,c1,c2,c3,c4**) scaffolds before (**a**) and after soaking in PBS for 7 (**b1,c1**), 14 (**b2,c2**), 21 (**b3,c3**) and 28 (**b4,c4**) days (*p < 0.05, **p < 0.01).

An ideal scaffold should not only have appropriate mechanical properties and cytocompatibility, but also be able to degrade at appropriate rate. Thus, the degradation behavior of the PLLA and PLLA/BN/ZnO-1-7 scaffolds was investigated in PBS solution, as shown in Fig. 7. The scaffolds before soaking in PBS had a flat, smooth and dense surface (Fig. 7a). The PLLA and PLLA/BN/ZnO-1-7 scaffolds could degrade in PBS and the degradation degree increased with the soaking time (Fig. 7b1–b4, [Fig f2], [Fig f3], [Fig f4],c1–c4, [Fig f2], [Fig f3], [Fig f4]). The PLLA scaffolds degraded faster than the PLLA/BN/ZnO-1-7 scaffolds and a large area of degradation was observed after 14 days of soaking or more time. It had 8.51% weight loss after 28 days of immersion, while the PLLA/BN/ZnO-1-7 scaffolds displayed a slower degradation rate with 7.17% weight loss during the same immersion time (Fig. 7d), which indicated that the addition of BNNSs and T-ZnO_w_ contributed a higher resistance to degradability of PLLA scaffolds. The result was in agreement with report by Şen *et al.*^38^, who found that the biodegradation of the gelatin-glucose scaffolds including BN was slower than the gelatin-glucose scaffolds without BN. Previous studies also showed that ZnO did not degrade in biological environment^39^, and could slow down the degradation rate^40^.

**Figure 8:**
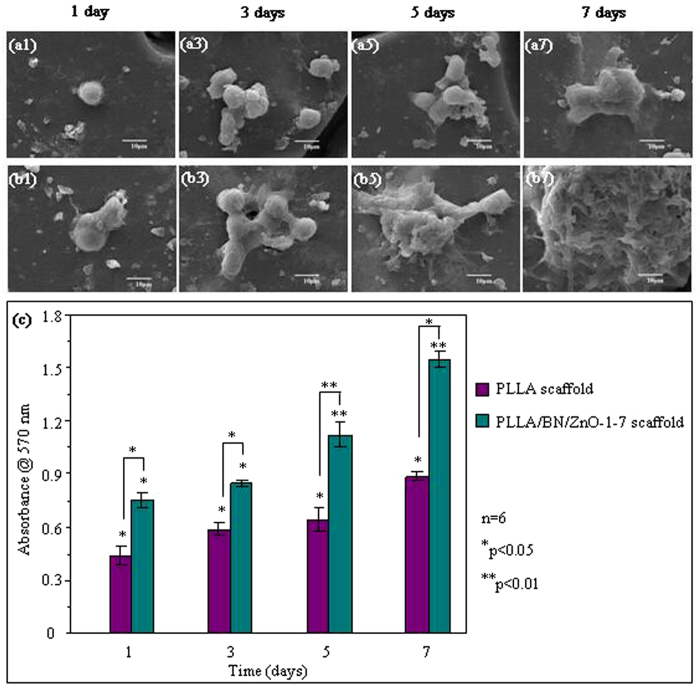
Attachment and proliferation of the hBMSCs cultured on the scaffolds for different periods. SEM images (**a1,a3,a5,a7,b1,b3,b5,b7**) and MTT assay (**c**) of the hBMSCs cultured on the PLLA (**a1,a3,a5,a7**) and PLLA/BN/ZnO-1-7 (**b1,b3,b5,b7**) scaffolds.

**Figure 9:**
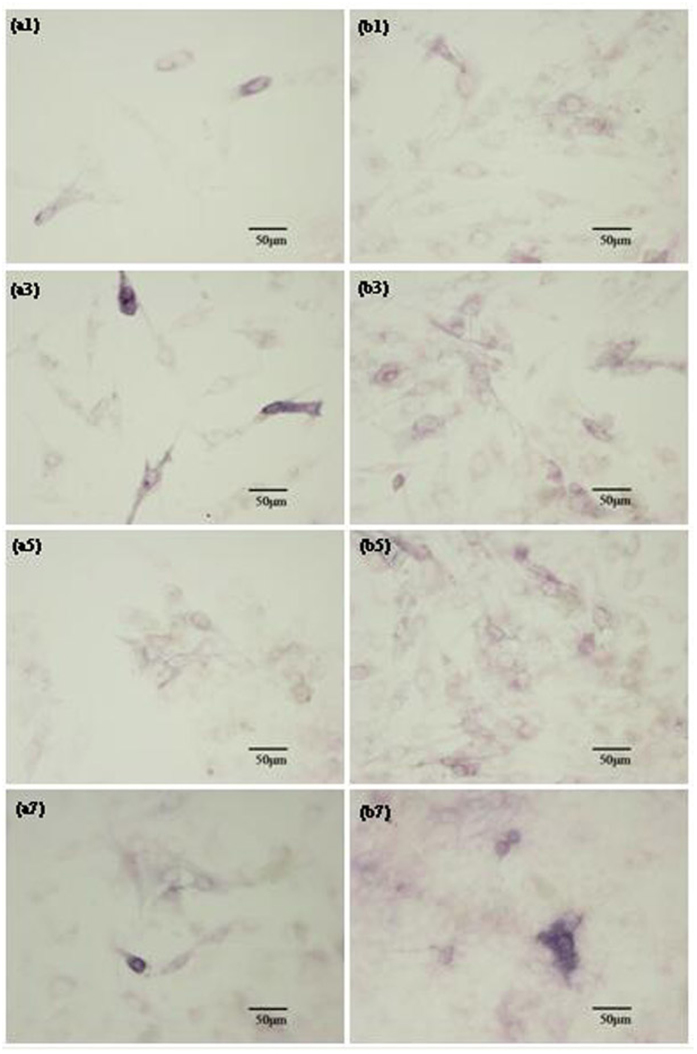
Osteoblastic differentiation of hBMSCs cultured on the scaffolds. ALP activities of hBMSCs cultured on the PLLA (**a1,a3,a5,a7**) and PLLA/BN/ZnO-1-7 (**b1,b3,b5,b7**) scaffolds for 1 (**a1,b1**), 3 (**a3,b3**), 5 (**a5,b5**) and 7 (**a7,b7**) days.

The attachment and proliferation of hBMSCs cultured on the PLLA and PLLA/BN/ZnO-1-7 scaffolds were determined by SEM analysis and MTT assay, respectively, as shown in Fig. 8. HBMSCs adhered on PLLA/BN/ZnO-1-7 scaffolds as well as the PLLA scaffolds and began to spread after 3 days of incubation. The cells on the two scaffolds proliferated over the culture time. The PLLA/BN/ZnO-1-7 scaffolds had a faster cell proliferation rate than the PLLA scaffolds, which indicated that the incorporation of BNNSs and T-ZnO_w_ into PLLA greatly facilitated hBMSCs proliferation. The differentiation of hBMSCs cultured on the PLLA and PLLA/BN/ZnO-1-7 scaffolds for 1, 3, 5 and 7 days was assessed in terms of ALP activity, as shown in Fig. 9. The ALP activity was increased in both PLLA and PLLA/BN/ZnO-1-7 scaffolds with increased incubation time. Similar to the cell proliferation results, the ALP activity of mesenchymal stem cells on the PLLA/BN/ZnO-1-7 scaffolds was higher than that on the PLLA scaffolds, indicating the significant up-regulated osteogenic differentiation of the cells.

## Discussion

Mechanical properties of scaffold are important factors in bone tissue engineering application because scaffold should withstand stress during culturing and implanting^41^. The mechanical properties of the PLLA/BN and PLLA/ZnO scaffolds were significant higher than that of the PLLA scaffolds (*p* < *0.05*). The results showed that the addition of BNNSs or T-ZnO_w_ enhanced the mechanical properties of PLLA scaffolds, while the enhancement efficiency was highly depended on the fillers content (Fig. 2). For example, the compressive strength, modulus and Vickers hardness of the PLLA/BN scaffolds increased with BNNSs content increasing from 0 to 0.75 wt% and then decreased with BNNSs content further increasing to 1.25 wt% (Fig. 2a,b). While the mechanical properties of the PLLA/ZnO scaffolds increased with T-ZnO_w_ content increasing from 0 to 5 wt% and then decreased with T-ZnO_w_ content further increasing to 9 wt% (Fig. 2c,d). The mechanical properties decreased due to the agglomeration of fillers. Previous studies demonstrated that the agglomeration of BNNSs or T-ZnO_w_ in polymer matrix decreased the mechanical properties^42^,^43^. The compressive strength, modulus and Vickers hardness of the PLLA/BN/ZnO-1-7 scaffolds were higher than that of the PLLA/BN/ZnO-0.75-5 scaffolds and about 96.15%, 32.86% and 357.19% higher than that of the PLLA scaffolds, respectively. A comparison of the increase in the strength, modulus and hardness of PLLA/BN/ZnO-1-7 scaffolds with PLLA composite with different reinforcement phase reported in the literatures^44^,^45^,^46^,^47^,^48^,^49^,^50^,^51^,^52^,^53^ are shown in Table 1. Although the scaffolds fabricated by SLS were porous structure, the mechanical properties laid in the same range as that of the composite fabricated by different method with different fillers. As we know, the mechanical properties decreased with the increase of porosity for porous materials^54^,^55^.

**Figure 10:**
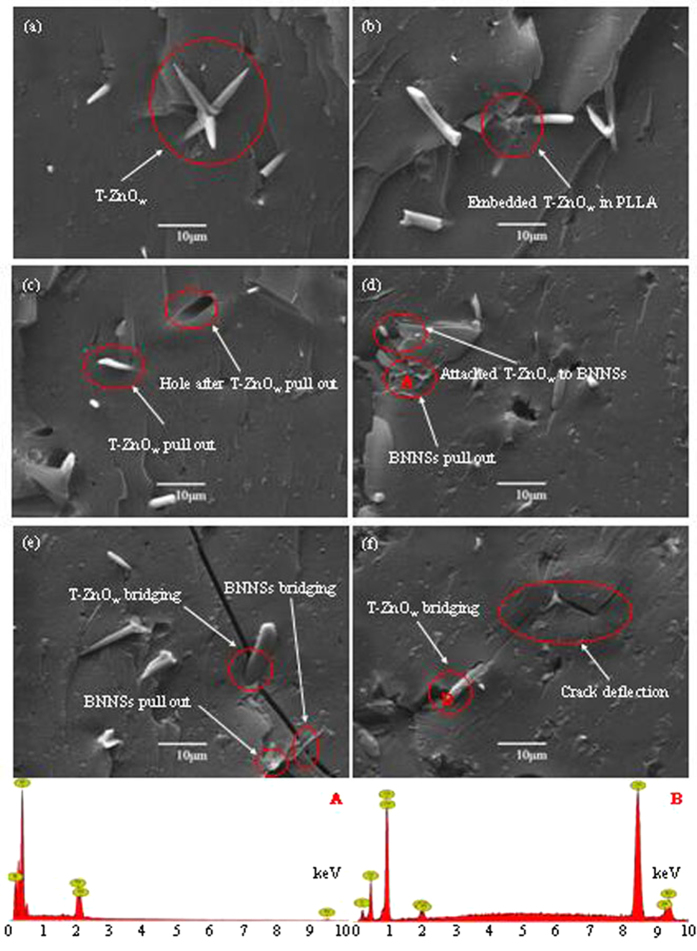
Fracture images and EDS spectra. Fracture images of the PLLA/BN/ZnO scaffolds (**a–f**), and EDS spectra of the element composite at the point A and B.

To investigate the reasons behind the mechanical properties enhancements, further studies were conducted to study the fracture images of PLLA/BN/ZnO scaffolds, as shown in Fig. 10. The fracture images revealed that the surface was quite smooth, which was the typical characteristic of brittle fracture behavior of PLLA^56^. T-ZnO_w_ with one needle pinning into the matrix and the other three needles pulled out from the matrix was observed (Fig. 10a). They were embedded in the PLLA matrix and only some of the needles could be observed (Fig. 10b). If one needle of the T-ZnO_w_ was pulled out from the matrix, the other three needles that embedded into the matrix had a role of anchor. Hole was observed because of the pullout of T-ZnO_w_ from PLLA matrix, indicating the strong interfacial interaction between T-ZnO_w_ and matrix (Fig. 10c)^57^,^58^. BNNSs pull out were visible, and some T-ZnO_w_ attached to BNNSs surface (Fig. 10d), which was an evidence for synergetic effect between BNNSs and T-ZnO_w_. BNNSs and T-ZnO_w_ could act as bridges and restrict the widening of crack (Fig. 10e), which increased the energy required to open the crack^59^,^60^,^61^. The propagation direction of the crack was altered and the crack deflection occurred when crack propagation encountered the T-ZnO_w_ (Fig. 10f). EDS analysis confirmed that the phase at point A and B were BN and ZnO, respectively.

It is known to us that cell behavior on the scaffold is influenced primarily by the chemical composition. Previous studies have already showed that PLLA could support osteoblast adhesion, spreading and growth^62^,^63^. In this study, the PLLA scaffolds could support MG-63 cell adhesion as well as migration, which suggested the good cytocompatibility of the material for bone scaffolds. Incorporation of BNNSs and T-ZnO_w_ into PLLA did not alter the major chemical composition. While introduction of Zn element into scaffolds is conducive to the improvement of osteoblast activity and bone formation^64^,^65^. In addition, it has been confirmed that BN possesses good cytocompatibility to human neuroblastoma cell, osteoblasts, macrophages, and so on^66^,^67^,^68^. For example, Lahiri *et al.*^67^ added boron nitride nanotubes (BNNTs) to biodegradable polylactide polycaprolactone (PLC) copolymer and found that PLC-BNNT composites exhibited increased osteoblast viability than that of PLC.

The attachment and proliferation of hBMSCs on the scaffolds of PLLA and PLLA/BN/ZnO-1-7 increased with culture time, indicating good cytocompatibility. Moreover, the proliferation of hBMSCs on the PLLA/BN/ZnO-1-7 scaffolds was significantly higher than PLLA scaffolds, indicating that the PLLA/BN/ZnO-1-7 scaffolds significantly promoted the cell proliferation. The ALP activity of hBMSCs cultured on the scaffolds increased with culture time, and the ALP activity of the cells on PLLA/BN/ZnO-1-7 scaffolds was significantly higher than PLLA scaffolds. The results revealed that the PLLA/BN/ZnO-1-7 scaffolds could promote cell osteogenic differentiation. It indicated that the addition of BNNSs and T-ZnO_w_ into PLLA scaffolds was beneficial to hBMSCs proliferation and differentiation, which played a critical role in new bone formation and growth. One possible reason for the improved cell proliferation and differentiation was that the addition of BNNSs into scaffold was beneficial for the expression of several essential cell adhesion proteins (such as fibronectin or vitronectin) during cell culture, which promoted cell adhesion and further increased cell proliferation and differentiation^69^,^70^. A recent study had shown that BN could result in an increase in the Runx2 gene expression level, indicating the increased osteogenic differentiation^67^. In addition, Zn ion could release from scaffold degradation and stimulate mesenchymal stem cells proliferation and differentiation into osteoblast^71^,^72^.

## Conclusions

In the presented work, two-dimensional BNNSs and three-dimensional T-ZnO_w_ were incorporated into PLLA matrix together to improve the comprehensive properties of scaffold fabricated by SLS. T-ZnO_w_ supported the interlayers of boron nitride in the direction perpendicular to BNNSs surface during the mixing process, helping their homogenous dispersion within the PLLA matrix. The optimal compressive strength, modulus and Vickers hardness of the scaffolds were obtained at a hybrid addition of 1 wt% BNNSs and 7 wt% T-ZnO_w_. Strengthening mechanisms were attributed to the formation of effective stress transfer between BNNSs, T-ZnO_w_ and the matrix due to BNNSs and T-ZnO_w_ pull out and bridging as well as the crack deflection. In addition, *in vitro* cell culture assays demonstrated that MG-63 cell and hBMSCs could attach, grow and spread on the scaffolds, and incorporation of BNNSs and T-ZnO_w_ together into PLLA scaffolds could promote MG-63 cell attachment and viability and enhance hBMSCs proliferation and differentiation. Overall, all these results indicated that the fabricated PLLA/BNNSs/T-ZnO_w_ scaffolds were promising candidates for bone tissue engineering.

## Materials and Methods

### Materials and reagents

PLLA was supplied by Jinan Daigang Biomaterial Co., Ltd. (Jinan, China) in powder form (M_w_ ∼ 10 kDa, T_g_ = 60–65 °C, T_m_ = 175–185 °C). BNNSs were provided by Nanjing XFNANO Materials Tech Co., Ltd. (Nanjing, China) (diameter: 0.5–5 μm, thickness: <50 nm, and purity: >99.5%). T-ZnO_w_ was obtained from Hefei Yijia New Materials Technology Co., Ltd. (Hefei, China) (needles length: 5–10 μm, needles diameter: 0.5 μm, and purity: >99%). MG-63 cells and hBMSCs were bought from the American Type Culture Collection (Rockville, MD). Dulbecco’s modified eagle’s medium (DMEM) and fetal bovine serum (FBS) were obtained from Gibco (CA, USA). 3-(4,5-dimethylthiazol-2-yl)-2,5-diphenyltetrazolium bromide (MTT) and dimethyl sulfoxide (DMSO) were purchased from Grand Island Biological Co. (Qingdao, China). All other chemicals and reagents were obtained from Sigma Aldrich (Beijing, China) and used as received.

### Preparation of the porous scaffolds

PLLA/BNNSs and PLLA/T-ZnO_w_ composite powder were prepared by ultrasonic dispersion followed by magnetic filtration, which involves (a) dispersing of PLLA/ethyl alcohol for 30 min with a SK3300H ultrasonic cleaning device (Shanghai Kudos Ultrasonic Instrument Co., Ltd., China), (b) adding appropriate amount of BNNSs and T-ZnO_w_ to the PLLA solution and continuing dispersing for another 30 min, respectively, (c) stirring the solution with a JB-5 magnetic stirrer (Jintan Ronghua Instrument Manufacture Co., Ltd., China), (d) filtering the solution and then drying overnight in 101-00S electrothermal blowing dry box (Guangzhou Dayang Electronic Machinery Equipment Co., Ltd., China). Similarly, PLLA/BNNSs/T-ZnO_w_ composite powder was also prepared using the same method.

Porous scaffolds were fabricated by selective laser sintering (SLS) using a 100 W CO_2_ laser (λ = 10.6 μm). The composite powder was sintered at the following optimized parameters: laser power of 3 W, scanning speed of 400 mm/min, spot diameter of 1 mm and bed temperature of 50 °C. The PLLA/BNNSs and PLLA/T-ZnO_w_ scaffolds were designated as PLLA/BN-x and PLLA/ZnO-y, respectively, and the PLLA/BNNSs/T-ZnO_w_ scaffolds were designated as PLLA/BN/ZnO-x-y, in which x and y were the weight percentage of BNNSs and T-ZnO_w_ content, respectively. For example, PLLA/BN/ZnO-1-7 means that the BNNSs and T-ZnO_w_ content in the scaffolds was 1 wt% and 7 wt%, respectively.

### Characterization of the scaffolds

The morphologies of the powder and scaffolds were observed using a FEI Quanta-200 scanning electron microscopy (SEM, FEI Co., USA). To observe the internal structures of scaffolds, the samples were frozen in liquid nitrogen for 8 min and then broken open using forceps. All specimens were sputter-coated with gold-palladium using a JFC-1600 auto fine coater (JEOL Ltd., Japan). The phase composition was determined by D8 Advance X-ray diffraction (XRD, German Bruker Co., German). The Cu-Kα nickel filtered radiation was detected at a 2θ angle range of 10–50 degrees. The compressive properties of scaffolds were measured using WD-D1 universal testing machine (Shanghai Zhuoji Instruments Co. Ltd., China) at a crosshead displacement speed of 0.5 mm/min. Six specimens (10 mm × 10 mm × 5 mm) were tested against each set. Vickers indentation tests were performed on the specimen (1 mm × 1 mm × 1 mm) surface to evaluate the Vickers hardness using a HXD-1000TM/LCD Vickers microindenter (Shanghai Taiming Optical Instrument Co. Ltd., China) with a testing force of 2.94 N and loading time of 15 s.

### Degradation behavior

The degradation behavior of the porous scaffolds was evaluated by measuring their weight loss in PBS according to the following procedure: The scaffolds (10 mm × 10 mm × 5 mm) were equally weighted placed in capped bottles containing 100 mL PBS, and then incubated in a rotary shaker (100 r/m) at 37 °C for different time intervals of 7, 14, 21 and 28 days. They were extracted at the end of each incubation period and washed three times with distilled water to remove ions absorbed on the surface, and then dried under electrothermal blowing dry box to constant weight. The weight loss (WL) was denoted as WL = (W_0_ − W_t_)/W_0_ × 100%, where W_0_ was the dry weight before degradation, W_t_ was the dry weight after degradation. The surface morphologies of the dried scaffolds after soaking for different times were determined with SEM.

### MG-63 cells adhesion and viability

MG-63 cells have similar characteristics of osteoblasts with the functions of synthesis, secretion and mineralization of bone matrix, making them attractive model for *in vitro* cell culture studies. The MG-63 cells were used to assay the cell adhesion and viability on the porous scaffolds of PLLA, PLLA/BN-0.75, PLLA/ZnO-5, PLLA/BN/ZnO-0.75-5 and PLLA/BN/ZnO-1-7 (10 mm × 10 mm × 5 mm). The cells were grown in DMEM supplemented with 10% FBS and 1% penicillin/streptomycin at 37 °C in a humidified 5% CO_2_ atmosphere. Prior to cell seeding, the scaffolds were sterilized in 70% ethanol and transferred to 24-well plates. MG-63 cells were seeded onto each scaffold at a density of 4 × 10^5^ cells. The culture medium was changed every other day.

The cell/scaffold constructs were taken out after 7 days of culturing and fixed in 2.5% glutaraldehyde for 20 min after incubation, and then washed with PBS three times and dehydrated in a series of graded ethanol for 10 min. They were gold-sputtered after drying in the dry box overnight and observed using SEM. The viability of MG-63 cells was studied using a live-dead fluorescence assay. The cell/scaffold constructs were rinsed with PBS three times after cell culture, and then exposed to 0.15 mm calcein-AM to target living cells and 2 mm ethidium homodimer-1 to target dead cells at 37 °C for 30 min followed by washing with PBS. Then they were analyzed using a fluorescence microscope (Olympus Co. Ltd., Tokyo, Japan) equipped with a digital camera (Olympus America Inc., Mel-ville, NY, USA).

### HBMSCs proliferation and differentiation

HBMSCs are bone marrow-derived mesenchymal stem cells, and they may differentiate into osteoblasts only under induction conditions. They were used to evaluate the inductive osteogenic differentiation ability of the bone scaffolds with the addition of BN and ZnO, while MG-63 osteosarcoma cells did not have the ability to evaluate the inductive osteogenic differentiation ability. The hBMSCs were seeded into a 24-well plate preloaded with the scaffolds (10 mm × 10 mm × 5 mm) at the concentrations of 5×10^5^ cells per scaffold and cultured at 37 °C in a humidified 5% CO_2_ atmosphere for 1, 3, 5 and 7 days. The culture medium was changed every other day. After cell culture, the cell/scaffold constructs were fixed in 2.5% glutaraldehyde, washed with PBS and dehydrated through a graded ethanol series, coated with gold and observed with SEM.

The hBMSCs proliferation on the scaffolds was quantitatively evaluated by MTT assay. 100 μl of MTT solution was added into each well after cell culture for 1, 3, 5 and 7 days and incubated at 37 °C for 4 h. And then 600 μl of DMSO was added to dissolve the reaction product formazan after removal of supernatants. Meanwhile, the blank wells of cell culture plate were used as the positive control. 200 μl of solution was transferred into a new 96-well plate and the absorbance was assessed at a wavelength of 570 nm using an enzyme-labeled instrument (Amersham, UK).

The inductive ability of the scaffolds was determined by alkaline phosphatase (ALP) expression using a LabAssay^TM^ ALP kit (Wako, Osaka, Japan) in accordance with the manufacturer’s protocol. The cell/scaffold constructs were gently rinsed with PBS after cell culture and incubated in 0.1% Triton X-100 solution for 10 min. And then, 100 μl of lysate containing 100 μl of *p*-nitrophenyl phosphate (*p*NPP) was added to the solution and incubated for 60 min. The ALP activity was assayed by measuring the release of *p*-nitrophenol from *p*NPP.

### Statistical analysis

Data from all studies were analyzed using SPSS Statistics version 19 (IBM Co., USA). Results were expressed as mean ± standard deviation (SD). *Denotes a significant difference when *P* < 0.05, and **denotes a very significant difference when *P* < 0.01.

## Additional Information

**How to cite this article**: Feng, P. *et al.* A space network structure constructed by tetraneedlelike ZnO whiskers supporting boron nitride nanosheets to enhance comprehensive properties of poly(L-lacti acid) scaffolds. *Sci. Rep.* **6**, 33385; doi: 10.1038/srep33385 (2016).

## Figures and Tables

**Table 1:** Comparison of mechanical properties achieved in scaffolds with literatures reported on PLLA composite with different fillers.

Filler and content	Processing method	Mechanical properties		
		Strength	Modulus	Hardness
1 wt% BNNSs + 7 wt% T-ZnO_w_ Present study	Selective laser sintering	34.15 MPa	2.83 GPa	243.18 MPa
50 wt% nano- or micro-HAP rods [ref. 44]	Thermally induced phase separation method	14.9 or 13.7 MPa	8.67 or 4.61 MPa	—
20–50 wt% HAP particles [ref. 45]	Forging process	106.7 ± 3.7~115.3 ± 3.9 MPa	5.3 ± 0.09~6.5 ± 0.2 GPa	20.8 ± 1.1~26.3 ± 2.2 MPa
1–10 wt% nanodiamond [ref. 46]	Solution casting and compression molding	114 ± 23~118 ± 15 MPa	4.4 ± 0.4~5.4 ± 0.5 GPa	—
1–10 wt% nanodiamond (ND) [ref. 47]	—	—	2.6 ± 0.1~7.9 ± 0.1 GPa	0.05 ± 0.01~0.46 ± 0.05 GPa
1–10 wt% octadecylamine-functionalized ND [ref. 48]	—	—	4.2 ± 0.1~5.1 ± 0.1 GPa	0.21 ± 0.01~0.26 ± 0.01 GPa
30 wt% magnesium [ref. 49]	Solvent casting and molding by compression	101.3 ± 4.6 MPa	8.01 GPa	340 ± 20 MPa
10–50 wt% chitosan microspheres [ref. 50]	Thermally induced phase separation method	1.42–1.63 MPa	15.4–25.5 MPa	—
3 wt% multi-wall carbon nanotubes [ref. 51]	Solution casting	19 MPa	235 MPa	—
1 wt% single-walled carbon nanotubes [ref. 52]	Solvent casting method	28 MPa	820 MPa	—
30 wt% ZnO whiskers + 10% carbon fibers [ref. 53]	Melt-mixing and hot-pressing	80 MPa	8.9 GPa	—
